# M’Gugin Monument

**Published:** 1867-12

**Authors:** E. R. Ford

**Affiliations:** Pres’t Board Curators, and Chm’n M Gugin Monument Assotiation


THE M'GUGIN MONUMENT.—As Chairman of the Committee of Arrangements appointed by the M'Gugin Monument Association, I take this opportunity of stating, for the benefit of the Profession and citizens, that the delay in the erection of the Monument proposed, has not arisen from any want of liberality on the part of the citizens or the Profession, but from the fact of the absence of myself and the President of the Association, Rev. Prof. E. J. Gelett, M. D., D.D., who were in charge of the work. I am happy to state that it has not been abandoned; that a portion of the funds has already been subscribed, and a contract entered into with Messrs. Maple & Stafford, of our city, for a suitable monument, which is now in progress, and will be completed within the next sixty days.

I now take this opportunity to appeal to the Profession, and especially to the *Alumni* of the Medical Department of the Iowa State University, who have so often sat beneath his teachings, and drawn from this fountain of science, to respond at once in this worthy enterprise, and aid in erecting to the memory of their loved and cherished Professor, a monument in commemoration of his worth while living, and in perpetuation of his memory while dead. We want from the Profession, for the completion of the monument, purchase of grounds and appropriate decorations, $1,000.

Send your remittances at once to the undersigned. The names of contributors, with the amounts subscribed by each, whether members of the Profession or citizens, will be placed in the List of Honor in the next issue of the JOURNAL, and the progress of the work fully set forth.

E. R. FORD, M. D.,

*Pres't Board Curators, and Chm'n M Gugin Monument Assotiation.*

